# Adjustment to Higher Education: A Comparison of Students With and Without Disabilities

**DOI:** 10.3389/fpsyg.2020.00923

**Published:** 2020-06-26

**Authors:** Orly Lipka, Miriam Sarid, Inbar Aharoni Zorach, Adi Bufman, Adi Anna Hagag, Hila Peretz

**Affiliations:** ^1^Department of Learning Disabilities, University of Haifa, Haifa, Israel; ^2^Edmond J. Safra Brain Research Center, Department of Learning Disabilities, University of Haifa, Haifa, Israel; ^3^Western Galilee College, Acre, Israel

**Keywords:** students, disabilities, higher education, adjustment, academic

## Abstract

The present study examined adjustment to higher education among students with disabilities from a multifaceted perspective (academic, social, emotional, institutional) immediately following their first year of study and onward, with three primary objectives. First, we examined whether students with no disabilities adjust better to higher education than do students with disabilities (mental, physical, sensory, ADHD/LD). Second, we examined differences among the specific disability groups in adjustment to higher education overall and in specific subscales. Finally, we examined the unique pattern of adjustment in each disability group, and sought to determine whether the groups differed with respect to this pattern. Of the 469 students who participated in the study, 234 had disabilities (mental disabilities, sensory, ADHD/LD, physical) and 235 were matched controls. The results indicated that students with disabilities as a whole reported lower adjustment than did controls. A close examination of the differences between the disability groups in the four subscales demonstrated unique adjustment challenges for each of them. The findings demonstrate the importance of specifically examining each disability group, to learn about needs and support.

## Introduction

Higher education, a principal means of achieving professional and economic goals, is seen by many today as a natural stage in the human life cycle. It is believed to contribute to self-determination and to the establishment of a positive self-image (Lidor et al., 2008). Post-secondary education, in particular, is a predictor of gainful employment in meaningful occupations, which open opportunities for career development and hence for good quality of life (Getzel et al., 2001; Dutta et al., 2009; Sachs and Schreuer, 2011).

College students with disabilities are a growing subpopulation at 2- and 4-year postsecondary institutions (Newman et al., 2011). This change is largely related to social and legislative policies and public opinions supporting the provision of equal education and employment opportunities for people with disabilities worldwide (Sarid et al., 2020). In Section 504 of the Rehabilitation Act of 1973, the United States government mandated postsecondary institutions to provide students with disabilities equal access to education, including support services.

Students with disabilities have varying levels of access to and support for grades K–16. Laws like IDEA (1975) ensure that students are provided with free and appropriate public education (FAPE) through high school or age 21. The 2004 IDEA Improvement Act specified that postsecondary institutions should be accessible and provide accommodations for students with disabilities. However, studies demonstrate that students with disabilities still face difficulties in these settings. Thus, despite the aforementioned rise in enrollment, in 2007, only one fourth of United States students with disabilities participated in some type of postsecondary education (Snyder et al., 2009).

Several studies have proposed theoretical models of retention and persistence in higher education (i.e., Tinto, 1975, 1993; Bean and Metzner, 1985), most of which focus on characteristics of students before entering post-secondary education that determine their persistence during the first year (Tinto, 1975, 1993). The majority of these studies examined students who represented the first generation in their families to attend post-secondary education (e.g., Warburton et al., 2001) and came from low SES backgrounds (e.g., Engle and Tinto, 2008). Few studies have examined theories of retention and persistence among students with disabilities (Kim and Lee, 2016).

### Higher Education in Israel

The Israeli higher education system has undergone dramatic changes since the mid-1990s. Initially based on six public research universities, it is now also encompassing over fifty public colleges, private colleges primarily focused on high-demand areas such as law and business administration, and teaching colleges. The central achievement of this structural reform was a rapid rise in the number of students enrolled in post-secondary education: from 75,000 in 1990 to approximately 265,000 in 2015 (CBS, 2015, Table 54.8). Today, nearly 50% of undergraduate students attend academic (public and private) colleges and 15% attend teaching colleges.

Alongside rising rates of enrollment in post-secondary education in Israel’s general population, a specific rise in students with disabilities has occurred. This is largely due to constitutional changes including the 1998 “Equal Rights for People with Disabilities Law,” which recognizes the rights of people with disabilities and the societal obligation to support and uphold these rights (Admon, 2007). Still, as in the United States, the proportion of Israelis with disabilities in post-secondary education remains lower (Ben-Simon et al., 2019) than the general population.

### Students With Disabilities

The Americans with Disabilities Act (ADA Amendments Act, 2008) defined disability as a physical or mental condition that causes substantial functional limitations of one or more life activities, including learning.

Learning disability (LD) and attention deficit/hyperactivity disorder (ADHD) are often classified as one group, “LD and/or ADHD,” in both research and clinical settings (DuPaul et al., 2013). In some cases, ADHD is categorized as a type of LD (Bizier et al., 2014). Explanations for this include high comorbidity rates, with the two disorders diagnosed comorbidity 45.1% of the time (DuPaul et al., 2013).

The number of students with LD enrolled in post-secondary institutions has increased dramatically. However, similar to students with ADHD, students with LD demonstrate difficulties in this context, with one study indicating that 80% of them had not graduated 5 years after high school, compared to 56% of students without disabilities (Murray et al., 2000). In addition, students with LD who display lower levels of adjustment to college reported greater need for counseling services and academic support (Saracoglu et al., 1989).

Another common disability in postsecondary education is mental disabilities. Those living with mental disabilities have lower chances of achieving their higher education goals and completing college (Collins, 2000) and approximately 86% of them will drop out of college before completing their degree (Collins and Mowbray, 2005). The enrollment numbers of students with hearing impairments in post-secondary education have also increased significantly over the past 25 years. One American study reports significant progress for deaf and hard of hearing students, citing an increase in overall enrollment in post-secondary education from 50% to 73% between 1990 and 2005. Physical disability is another condition that influences the adjustment of students to postsecondary education.

### Hidden Disabilities

Hidden disabilities are defined as “conditions that provide no atypical appearance or no readily observable functional limitations to those interacting with the individual peripherally” (Falvo et al., 1982). Though not as vulnerable to stigmatization due to overt differences, individuals with invisible disabilities can experience employment difficulties, presumably due to anxiety related to having an invisible disability (Frable, 1993). Across the United States, there is evidence that approximately 10% of undergraduate and graduate students report having a disability. Approximately 70% of these students reported disabilities such as learning, attention, psychiatric, or chronic health impairments (Raue and Lewis, 2011). Students with ADHD/LD or mental disabilities face obstacles that moderate their success in higher education, such as psychological distress, poor social and interpersonal skills, persistent cognitive deficits (especially in the area of executive functioning), and alcohol abuse (Wolf, 2001), or attentional difficulties (American Psychiatric Association [APA], 2013), compared with other students with disabilities. ADHD is classified in DSM-5 as a mental disabilities (American Psychiatric Association [APA], 2013).

Visible disabilities are more stigmatized than invisible disabilities. Indeed, in an empirical study utilizing multidimensional scaling, Frable found that visibility was a “master status” that causes people to be treated differently (Frable, 1993). Some students with disabilities choose not to disclose their disabilities, to mitigate stigmatization, and as a result receive less academic support from the academic institution (Grimes et al., 2017).

### Adjustment

A key factor in determining whether students remain and succeed in post-secondary education is their ability to adjust to the often complex environment (“post-secondary adjustment”). Students who experience difficulties in adjusting to the academic environment are at higher risk of attrition (Adams and Proctor, 2010).

In the past, investigators viewed post-secondary adjustment as a single variable (Mooney et al., 1991, p. 445). The more current, multifaceted view suggests that academic adjustment comprises functioning in four distinct domains. The first, “academic achievement,” includes motivation for learning, appropriateness of skills to academic requirements, and ability to earn satisfactory grades. The second domain, “social adjustment,” encompasses involvement in the study environment, including the ability to establish social networks. The third, “personal emotional adjustment,” reflects psychological and physical conditions; it is indicative of self-perception and represents the ability to cope with study-related challenges that can lead to stress and anxiety. The fourth and final domain, “institutional adjustment,” involves how students feel about their relationship with academics, in general, and to their academic environment, in particular (Baker and Siryk, 1984, 1989; Gerdes and Mallinckrodt, 1994).

Many studies have examined adjustment among students in post-secondary education, especially during the first year of study (i.e., Horn and Carroll, 1998). Most of the research conducted in this field addresses the first year of post-secondary education, in which most of the dropouts occur. The research on adjustment of students in the second and third years of study is limited and the characteristics of adjustment in this group are largely unknown. Among the variables that have been examined is psychological capital (i.e., Liran and Miller, 2019). Liran and Miller, for example, found that psychological capital, as manifested in four variables, self-efficacy, hope, optimism, and resilience, predicted adjustment to academic studies in a sample of 250 typical second and third year students at an Israeli university.

### Adjustment in Students With Disabilities

Some research has examined students at higher risk for adjustment difficulties, such as international students (i.e., Poyrazli and Grahame, 2007; Lashari et al., 2018; Lee et al., 2018). Another group of studies examinedthe adjustment of students with disabilities. In some cases, researchers examined a group of students with disabilities as one group, such as LD, ADHD, physical, mental disabilities, or sensory difficulties (Murray et al., 2014). Though limited, the work on students with disabilities suggests that they are indeed at risk in terms of overall adaptation to the college experience, social adjustment, and institutional attachment to college (Adams and Proctor, 2010), and that they can experience logistical, socio-environmental, and attitudinal barriers to success (Wolanin and Steele, 2004). Krisher and Shechtman (2016), for example, showed that the academic achievement and adjustment scores of students who reported having LD were lower than those of students who did not. Murray et al. (2014) measured adjustment by means of self-efficacy to college, student engagement, and GPA. They revealed three distinct adjustment profiles of students, and approximately twice as many students with disabilities were classified as poorly adjusted as were classified as highly adjusted (Murray et al., 2014).

It appears that students with disabilities in postsecondary education face the same challenges as do students without disabilities, with the addition of challenges specifically related to their disabilities. Several studies have compared students with and without disabilities with respect to college adaptation using the Student Adaptation to College Questionnaire (SACQ; Baker and Siryk, 1984), which assesses overall adjustment to college, as well as the four specific areas of academic adjustment, personal–emotional adjustment, social adjustment, and attachment (to the institution). While students with and without disabilities were not found to differ in terms of academic achievement, they did differ with respect to the subscales measuring social adjustment, personal-emotional adjustment, and school attachment. Students with disabilities were significantly less adapted than their peers without disabilities on all three of these psychosocial dimensions (Adams and Proctor, 2010; Herrick, 2011). This finding, reported in two separate studies, indicates greater struggles with psychosocial aspects of adaptation to college among students with disabilities (Adams and Proctor, 2010; Herrick, 2011). In addition, higher levels of self-reported visibility of disability to others and better self-advocacy skills were statistically significant positive predictors of adaptation to college (Adams and Proctor, 2010).

Another limited group of studies examined adaptation to post-secondary education among specific groups with disabilities. While an increasing number of students with ADHD now attend institutions of higher education (Green and Rabiner, 2012), they appear to struggle throughout their academic career, and their chances of graduating are significantly lower than those of students without disabilities (Weyandt and DuPaul, 2006). In accordance, students with ADHD were shown to have significantly lower scores on the SACQ than their peers without ADHD. Students with ADHD also had lower levels of self-reported social skills and self-esteem, both of which were associated with lower levels of adaptation to college (Shaw-Zirt et al., 2005).

One study reported fewer experiences in the three activities related to social inclusion, namely art and theater events, clubs and organizations, and student acquaintances, among students with psychiatric disability. These students also estimated gains from their studies as lower than those of the two other groups. On the other hand, students with psychiatric disability were more likely to participate in library activities than students with physical and sensory disabilities. Students with physical disabilities were more satisfied with their studies than were the other two groups of students with disabilities (Sachs and Schreuer, 2011).

### The Current Study

Limited studies have examined the adjustment of specific disability groups from a multicomponent perspective beyond the first academic year. Therefore, to address the paucity of research on adjustment to postsecondary education among students with disabilities, and to focus on single disabilities, the present study examined the impact of four unique disabilities that vary with respect to visibility and mental disabilities, as well as physical and academic impairment, in a large sample. The aim was to gain a deeper understanding of adjustment to higher education among groups of students with different disabilities, as compared to one another and to a typical control group, after the first year of study and onward. In addition, the study aimed to examine the adjustment of students with visible versus invisible disabilities. A broad and diverse sample of Israeli post-secondary (i.e., university or college) students was employed alongside a control group matched for gender, academic faculty, institution, and year of study.

The study had three specific objectives. First, we examined adjustment among all the students with disabilities in our sample, compared with the control group. Second, we examined social, academic, institutional, and emotional components of adjustment among the four disability groups (LD/ADHD, physical, mental, and sensory). Finally, we examined the unique pattern of adjustment in each group of students with disabilities, and evaluated differences between the groups in an attempt to shed light on their specific needs and challenges. We predicted that students with no disabilities would have higher adjustment scores than students with disabilities, and students with difficulties related to academic performance would have the lowest adjustment scores.

## Materials and Methods

### Participants

Table 1 presents the demographic and background characteristics of the sample. Overall, 469 college and university students from eight universities and fifteen colleges in Israel participated in the study, of which 235 (68% female) had disabilities and 232 (75% female) did not. Of the participants, 95% were undergraduates and 5% were graduate students; Hebrew was the first language of 94% of the students.

**TABLE 1:** Demographic and background characteristics of the sample.

		Students with disabilities		Control		
		*n*	%	*n*	%	Chi Sq.
Gender	Male	76	32%	59	25%	2.52
	Female	161	68%	173	75%	
Type of institution	University	155	65%	154	66%	0.05
	College	82	35%	78	34%	
Have you studied in another post-secondary institution in the past?	Yes	51	22%	33	14%	4.24*
	No	186	78%	199	86%	
Emotional/academic/social support from the academic institution	Yes	129	54%	65	28%	33.7***
	No	108	46%	167	72%	
Have you been diagnosed with emotional or mental disability?	Yes	199	84%	6	3%	316.5***
	No	38	16%	226	98%	
Received support from the National Insurance Institute?	Yes	66	28%	1	0%	
	No	171	72%	231	100%	
Do you work?	Yes	159	67%	178	77%	8.86*
	No	73	31%	54	23%	
	Scholarship	5	2%	0	0%	
Do you have support from other role holders in the institution?	Yes	46	19%	25	11%	6.80**
	No	191	81%	207	89%	

*p < 0.05; **p < 0.01; ***p < 0.001.

Four disability subtype groups were based on participant self-reports, and included hidden and visible disabilities: hidden disabilities included mental disabilities (*n* = 63) and ADHD/LD (*n* = 67), while visible disabilities included sensory (*n* = 53) and physical disabilities (*n* = 54). For each group of students with disabilities, a group of students without disabilities from the same institution was matched with respect to gender, field of study, and year of study. About 25% of the participants in the mental disabilities group, 12% of the ADHD/LD group, 17.5% of those with sensory disabilities, and 29% of those with physical disabilities resided on campus. In the control group, 30% reported that they reside on campus. Chi square comparison indicated a significant difference in the ratio of those who reside on campus among the groups (Chi square = 9.8, *p* = 0.04). About 54% of the students with disabilities received some kind of support from their academic institution, while no differences were found with regards to the ratio of support between groups of disabilities (Chi square = 0.11, *p* = ns). In addition, no differences were found in the type of support the participants received. The main support types reported were academic (40% of the participants with disabilities), financial support (13%), and emotional support (19%). The groups did not differ in the type of supports received (Chi square = 37.1, *p* = ns).

To address the possibility of comorbidity of symptoms between disabilities, the participants were asked to note the disability most significantly affecting their functioning.

### Measures

#### Background and Demographic Questionnaire

The questionnaire included demographic information regarding gender, year, field, and institution of study, and diagnosis of disability.

#### Student Adaptation to College Questionnaire

The original Student Adaptation to College Questionnaire (SACQ) was a 52-item self-report questionnaire. It was developed in English and translated to Hebrew by Khalili (2006) using the standard back translation method. Participants respond to each of the questionnaire items on a Likert scale ranging from 1 (doesn’t apply to me at all) to 9 (applies to me very much), such that higher scores represent better adjustment. A shortened Hebrew modified version of the original SACQ (Baker and Siryk, 1989) was developed and partly modified in order to adapt the content to the reality of Israeli university and college students.

The questionnaire comprises four subscales: academic adjustment, including 19 items addressing adaptation to academic challenges in the learning environment (e.g., “Lately I have been having doubts regarding the value of a college education”), with high internal consistency, Cronbach’s alpha = 0.87; personal-emotional adjustment, including 9 items addressing general psychological distress (e.g., “I have been feeling tense or nervous lately”), Cronbach’s alpha = 0.81; social adjustment, including 12 items addressing the ability to cope with social interactions (e.g., “I am meeting as many people and making as many friends as I would like to the college”), with high internal consistency, Cronbach’s alpha = 0.83; and institutional adjustment, including 6 items addressing commitment to academic goals (e.g., “I am happy with my decision to attend this college”), Cronbach’s alpha = 0.71.

In addition, data were collected regarding emotional/social and academic support services used by participants, and the period of time such support was received.

### Procedure

Support offices at most institutions of higher education in Israel (all the universities, many colleges, and a teachers’ college) were asked to send their registered students an online post about the study and an electronic questionnaire. The current study was part of a larger study that examined different variables contributing to the adjustment of students in higher education. Data were collected over 2 years. Students with disability who completed the questionnaires were asked to pass them on to a friend at the same college with no diagnosed disability, matched for faculty and department, gender, and year of study. When students with disability were not able to find a matched control, a request was posted on the Facebook page of the institution or by the department secretary. Students returned the questionnaires to the researcher via email and received $20 for their effort. Prior to entering the study, participants were given an explanation regarding its purpose and procedure, and signed an informed consent form. This research was supported by the Israeli National Insurance Institute (social security). In addition, the study received the approval of the University of Haifa Faculty of Education ethics committee.

### Data Analysis

SPSS version 25 was used to analyze the data. To address the first research question, differences between the study and control groups were tested using an independent samples *t*-test. To address the second research question, a comparison between the four groups of disabilities and the control group was conducted using multivariate analysis of variance (MANOVA), followed by univariate analyses of variance and Tukey *post hoc* pairwise comparisons. Finally, to examine the unique pattern of adjustment in each group of students with disabilities, the differences between adjustment subscales within each group and between the groups were tested with a two-way repeated measures MANOVA with Adjustment Subscale as a within-subjects factor and Group as a between-subjects factor (4 × 5). Following this general comparison, we conducted pairwise comparisons in each group, with Bonferroni corrections to account for cumulative type 1 error. To better understand the adjustment patterns, we classified the participants into clusters using a k-means cluster analysis with the four adjustment subscales. The number of clusters was determined according to the ‘elbow’ method, in which the number of clusters for k-means was chosen by fitting k-means models for a range of consecutive numbers, usually 1 up to some maximum number, and plotting an elbow plot of the total within sum of squares (WSS) value for each number of clusters versus that cluster number (Flynt and Dean, 2016). In addition, cross-tabulations and chi square tests were used to compare the groups on demographic variables. Chi square test as well as cross-tabulation was also used to compare the cluster distribution in each group of participants, and also to compare visible versus invisible disability groups.

Pearson correlations were used to examine the correlation between subscales of adjustment.

## Results

The first aim of the study was to compare the adjustment scores of the entire sample of students with disabilities to those of the control students without disabilities. To this end, an independent samples *t*-test was conducted with total adjustment score as the dependent measure. The results indicated lower adjustment scores in students with disabilities (*M* = 5.90, *SD* = 1.11) as compared to controls (*M* = 6.57, *SD* = 0.96), *t*(467) = 6.88, *p* = 0.001, *d* = 0.60.

The second aim was a detailed examination of adjustment among the five study groups (four disability groups and typical students), based on the four adjustment subscales. A one-way multivariate analysis of variance (MANOVA) was conducted, followed by univariate analyses of variance (ANOVAs) for each adjustment measure, and *post hoc* Tukey tests.

**FIGURE 1:**
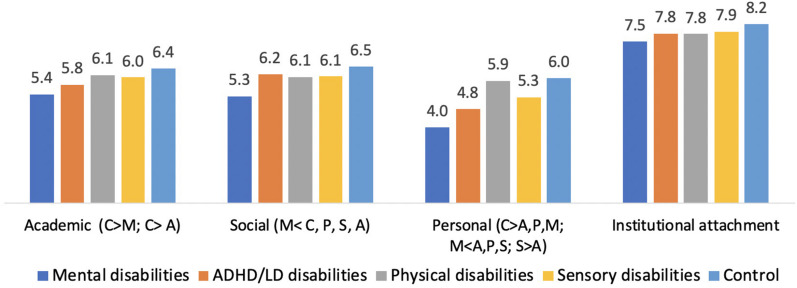
Adjustment to higher education by category of adjustment and group of disability. C, control group; A, attention-deficit/hyperactivity disorder/learning disabilities (ADHD/LD) group; M, mental disabilities; P, physical disabilities; S, sensory disabilities.

A main multivariate effect was found between the groups with respect to adjustment level, Multivariate Wilks’ *F*(16,1409) = 7.70, *p* < 0.001, *η* = 0.06. As shown in Figure 1, the results indicated that the scores of students with disabilities were lower than those of their peers without disabilities on all four adjustment subscales. Pairwise comparisons revealed the following results:

The *academic adjustment* of students with mental disabilities was lower than that of the following three groups: sensory disabilities, physical disabilities, and controls. Students with ADHD/LD had lower academic adjustment scores than controls but did not differ significantly from the other disability groups.

The *social adjustment* scores of students with mental disabilities were lower than those of students with other disabilities, and of typical students. No other between-group differences were found with respect to social adjustment.

*Personal adjustment* scores were lower in the mental disabilities group than in all other disability groups and the control group. The sensory disability group’s adjustment scores were higher than those of the ADHD/LD group, and personal adjustment score was lower than that of control group.

The control group showed higher institutional adjustment scores than the mental disabilities group. No other between-group differences were found with respect to institutional adjustment.

In addition to differences, as can be seen in Table 2, Pearson correlation coefficients were calculated between the adjustment subscales. All the correlations were found to be higher than *r* = 0.36 and significant (*p* = 0.001), in the entire sample as well as in each group of students with disabilities.

**TABLE 2:** Pearson correlation coefficients between adjustment subscales.

	Academic	Social	Personal
Social	0.60		
Personal	0.63	0.51	
Institutional	0.60	0.53	0.44

The third research objective was to identify the unique pattern of adjustment in each group of students with disabilities and examine whether the groups differed with respect to this pattern. We began with a two-way repeated measures MANOVA with Adjustment Subscale as a within-subjects factor and Group as a between-subjects factor (4 × 5). The results indicated a significant Group x Adjustment Subscale interaction with respect to academic versus institutional adjustment, *F*(4,464) = 2.48, *p* < 0.05, and personal versus institutional adjustment, *F*(4,464) = 14.72, *p* < 0.001. Following this general comparison, we conducted pairwise comparisons in each group, with Bonferroni corrections to account for cumulative type 1 error (see Table 3 and Figure 1).

**TABLE 3:** Means (M), standard deviations (SD), and ANOVA results examining differences in adjustment measures between groups of students with and without disabilities.

Adjustment subscale		Mental disabilities *n* = 63	ADHD LD *n* = 67	Sensory disabilities *n* = 53	Physical disabilities *n* = 54	All students with disabilities *n* = 237	Control *n* = 232	*F df* = 4,464	ηp^2^
Academic	*M*	5.36	5.76	6.14	6.04	5.80	6.40	12.1***	0.09
	*SD*	1.21	1.03	1.26	1.18	1.20	1.12		
Social	*M*	5.30	6.16	6.07	6.07	5.89	6.47	10.4***	0.08
	*SD*	1.49	1.26	1.48	1.47	1.46	1.14		
Personal	*M*	4.04	4.78	5.91	5.26	4.95	6.00	28.5***	0.20
	*SD*	1.31	1.52	1.64	1.36	1.60	1.43		
Institutional	*M*	7.48	7.81	7.77	7.88	7.73	8.18	5.62***	0.05
	*SD*	1.44	1.18	1.36	1.36	1.33	0.90		
Adjustment	*M*	5.37	5.94	6.29	6.13	5.91	6.57		
(Total score)	*SD*	1.04	0.99	1.19	1.05	1.11	0.96		

ADHD, attention-deficit/hyperactivity disorder, LD, learning disability. Multivariate Wilks’ F(4,464) = 14.8, p < 0.001, ηp = 0.11, ***p < 0.001.

*Students with mental disabilities*. Personal adjustment was the subscale with the lowest scores, as compared to the other three subscales, among participants with mental disabilities. Institutional adjustment had the highest scores compared to the other adjustment types.

*Students with ADHD/LD*. We found significant differences between all adjustment subscales in this group, with institutional adjustment receiving higher scores than social, academic, and personal adjustment.

*Students with sensory disability*. Institutional adjustment scores were higher than those of all other subscales among the students with hearing and vision impairments. No other differences were found between the subscales.

*Students with physical disability*. As in the other groups, institutional adjustment scores were higher than other adjustment scores in students with physical disabilities. In addition, their personal adjustment scores were lower than their social and academic adjustment scores.

*Control group*. Like participants with physical disabilities, controls had the highest scores on institutional adjustment, and lower personal adjustments scores than academic and social adjustment scores.

To better understand the adjustment patterns in the different disability groups (third research question), we conducted a k-means cluster analysis with the four adjustment subscales in the whole group of participants. The analysis classified the entire sample into two clusters (groups); The number of clusters was determined based on the “elbow” method. According to this method, the number of clusters for k-means was chosen by fitting k-means models for a range of consecutive numbers, usually 1 up to some maximum number, and plotting an elbow plot of the total within sum of squares (WSS) value for each number of clusters versus that cluster number (Flynt and Dean, 2016). The value for one cluster was WSS = 3358.3, and the value for two clusters was WSS = 1801.9. For three clusters the value dropped to WSS = 1423, and therefore, two clusters were chosen as the number of clusters.

**FIGURE 2:**
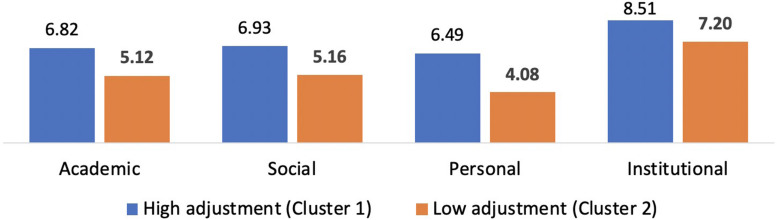
Students with disabilities: adjustment mean scores of high and low adjustment students.

Following the cluster analysis, the group means were examined and revealed that the first cluster (see Table 3) represented respondents who had higher scores on all adjustment subscales (*n* = 270) and the second cluster represented respondents who had lower scores on all adjustment subscales (*n* = 199). To further investigate clusters in the groups, in the next step we cross-tabulated the groups by cluster (see Table 4 and Figure 2).

**TABLE 4:** Distribution of clusters in the groups and mean adjustment subscale scores according to cluster analysis.

		High adjustment (Cluster 1) *n* = 270	Low adjustment (Cluster 2) *n* = 199	Statistical differences
Adjustment subscale	Academic	6.82 (0.81)	5.12 (0.91)	*F*(1,467) = 453***
	Social	6.93 (0.87)	5.16 (1.19)	*F*(1,467) = 345***
	Personal	6.49 (1.10)	4.08 (1.04)	*F*(1,467) = 577***
	Institutional	8.51 (0.57)	7.2 (1.32)	*F*(1,467) = 212***

***p < 0.001.

**FIGURE 3:**
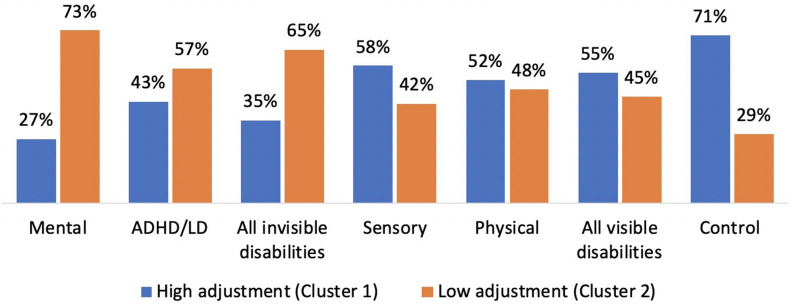
Ratio of low and high adjustment students in groups of disability.

The results of the cross-tabulation showed that the majority of participants with mental disabilities or ADHD/LD were classified in the low adjustment group, whereas the majority of participants in the control and sensory disability groups, were classified in the high adjustment cluster (for means of adjustment subscales by cluster see Figure 3).

Participants with physical disability were divided approximately half and half. We then divided the groups into visible (physical and sensory disabilities) versus invisible disabilities (mental disabilities and ADHD/LD disabilities), and repeated chi-square analysis. Results showed that in the invisible disabilities group, a higher percentage of students were classified in the low cluster, while in the visible disabilities group, a higher percentage were classified in the high cluster. An additional chi-square subgroup analysis indicated significant differences between each group of either visible and invisible disabilities versus control (χ^2^ = 8.34, *p* = 0.01; χ^2^ = 43.8, *p* < 0.001) and also between those two groups of disabilities (χ^2^ = 9.28, *p* < 0.01).

## Discussion

While post-secondary education is associated with academic, social, personal, and institutional challenges for all students, students with disabilities are likely to face additional challenges. The current research examined adjustment in students with disabilities attending institutions of post-secondary education in Israel. Participants came from a representative sample including students with one of four specific disabilities, mental disabilities, sensory, physical, or ADHD/LD, and students without disabilities.

The first research question examined differences between all students with disabilities and control students with respect to general adjustment. The findings showed that, as a group, students with disabilities adapted less successfully than students without disabilities. These findings are in line with those of a similar study by Adams and Proctor (2010), who referred to adaptation as a univariate measure. Given that adjustment is related to dropout rates and that students with disabilities are at particular risk for dropping out, attention to their adjustment needs is important (Costello and Stone, 2012).

Our second aim was to compare the four groups of students with disabilities to typical students with respect to the four adjustment subscales. Overall, students with disabilities reported lower adjustment compared to their peers without disabilities on all four measures. These results are generally consistent with most previous findings on adjustment among students with disabilities (Adams and Proctor, 2010; Herrick, 2011).

While previous studies found significant differences between students with and without disabilities on three subscales (social, personal, and institutional), the current study suggests that these difficulties extend to academic aspects of adjustment as well. This difference might be due to differences in the characteristics of the disability groups examined. In accordance, the current study shed light on difficulty patterns (social, personal, institutional, academic) associated with specific disability groups, which can serve as the basis for the provision of tailored support.

Students with mental disabilities demonstrated lower adjustment scores on all four subscales, reporting lower personal and social adjustment compared to all the other disability and control groups. Their lower scores on personal adjustment are not surprising, taking into consideration their type of disability. Furthermore, this finding might validate their self-classification into a group with mental disabilities. This group also reported greater difficulties with academic adjustment than the physical and sensory disability groups and had significantly lower institutional adjustment scores than did controls. These results are partially consistent with previous findings on students with mental disabilities. Collins and Mowbray (2005), for example, reported that this group experienced difficulties with concentration, time management, class attendance, coping with stress, non-completion, poor physical health, and building social networks. Furthermore, studies have shown that students who experience mental disabilities have lower completion rates than other students with disabilities (Martin, 2010). In keeping with previous results, the current study highlights the constant challenges that students with mental disabilities experience in many aspects of post-secondary education and calls for support services tailored to meet these challenges.

Students with ADHD and/or LD are another group of students that reported significant difficulties in several aspects of adjustment. As expected, this group demonstrated significantly lower scores on academic and personal adjustment compared to the control group. The current results are therefore consistent with previous reports that ADHD is associated with lower grade point average, more academic difficulties, and less effective study skills (Advokat et al., 2011; Weyandt and DuPaul, 2013; Gormley et al., 2015). Our results also support Tinto’s (1975) model and proposition that student trajectories and, in turn, academic persistence are shaped by the interaction between experiences in high school and at college (Tinto, 2010). Students with ADHD/LD are likely to experience academic difficulties during their earlier school years, which might influence their academic adjustment during post-secondary education.

Previous work has also shown that students with ADHD and/or LD report more symptoms of depression and anxiety than non-ADHD/LD students do (Hoy et al., 1997; Carroll and Iles, 2006; Rabiner et al., 2008; Blase et al., 2009). The results of the current study support these findings as well, as students with ADHD and/or LD reported difficulties in personal adjustment (e.g., “I have been feeling tense or nervous lately”). This finding has implications for the type of support services that should be provided for these students and calls for close monitoring during their post-secondary journey. ADHD is classified in the DSM-5 (American Psychiatric Association [APA], 2013) as a mental disability. Nevertheless, the current study results that revealed differences between ADHD and those who self-identified as having a mental disability, provide empirical justification for differentiating between these groups. The symptoms of anxiety, depression, and attention deficits may affect adjustment and functioning in higher education, and are less typical among students with visible disabilities such as motor or other physical disabilities.

Students with physical disabilities only showed lower adjustment than controls in the personal adjustment category, and most of the adjustment difficulties reported by students with sensory disabilities were in the personal realm as well. There is very limited research on these two groups in the context of adjustment to higher education, such that future studies are warranted to reveal the particular challenges they face. One implication of this result is that institutions should identify students with invisible disabilities early on, probably during the registration stage, examine their adjustment needs (social, emotional, and academic), and provide them with appropriate support.

Our third research question addressed the unique pattern of adjustment in each group of students with disabilities, and examined whether the groups differed with respect to this pattern. For all groups, including controls, institutional aspects of adjustment seemed to be the least problematic. Institutional adjustment reflects academic commitment to the institution and, indirectly, student retention. It is well-documented that students who drop out of postsecondary education mostly do so during their first year (Chen, 2012; Respondek et al., 2019). As the participants in the current research were all in their second year or beyond, they were likely to be more committed to their studies and to the institution they attend. We can therefore conclude that second and third year students face fewer institutional adjustment difficulties than other types of adjustment difficulties.

In the control group, personal adjustment appeared to be more problematic than social, academic, and institutional adjustment. This pattern was also revealed in the two invisible disability groups (i.e., mental disabilities and ADHD/LD) as well as the physical disability group. Personal adjustment might be a reflection of daily stress and concerns inherent in the academic environment, such as stress resulting from academic requirements or from daily conflicts between academic and work demands.

Our ANOVA results were supported by a clustering procedure in which all the students were divided into low and high adjustment clusters, independent of disability. The classification of these clusters indicated a higher percentage of students with low adjustment in the invisible disabilities groups, and a lower rate of low adjustment students in the visible disabilities group. It should be noted that clustering of students was conducted according to their perception of adjustment, and therefore the clustering reflects severity of adjustment and not the disability itself. Self-perception can indicate the student’s functioning in postsecondary education, and thus might be no less important than objective severity of disability. These results are supported by a similar result presented by Murray et al. (2014) which showed over-representation of students with disabilities in a low adjusting group, as approximately twice as many students with disabilities were classified as low in adjustment than were classified as high.

In addition, it is possible that the group of those with hidden disabilities was less inclined to disclose their disabilities in order to be perceived merely as students and not as disabled, as proposed by Grimes et al. (2017): “Choosing non-disclosure may offer an opportunity to re-develop their identity away from “disabled” and toward “university student.” However, these students might still have more adjustment difficulties than others.

The current study had several limitations. First, we mostly used self-report measures, which are based on subjective perceptions. Some of our disability groups include comorbid disabilities, which probably reflect the actual population at the postsecondary education level. These comorbidities should be taken into consideration in interpreting the findings. Future studies should include more objective measures, such as grade point average and dropout rate. In addition, we examined specific disability groups. It is necessary to extend the use of the Student Adaptation to College Questionnaire (SACQ; Baker and Siryk, 1989) to different types of students with disabilities. Future studies should include additional groups, such as students with autism, who have been shown, for example, to struggle during the transition from high school to post-secondary education (e.g., Kapp et al., 2011), and more information on the adjustment of these students and students with different disabilities is needed. In addition, this study investigated adjustment beyond the first year of studies and therefore does not relate to challenges that first year students may face. As a high proportion of students with ADHD/LD do not participate in postsecondary education, our participants may not have had the same level of academic difficulties as others who do not study in college, which might be related to their adjustment. This issue requires further investigation of additional academic parameters such as college achievements and dropout rates.

Even within the broad categorization of disability employed in this study, closer examination of specific subtypes will provide a better understanding of adjustment. For instance, students with physical disabilities in the current study included a wide variety of causes, such as chronic disease, amputation, and more. Each condition could manifest in a different way and might require different types of support.

One of the findings revealed lower personal adjustment of the group with mental disabilities. This finding is not surprising, as this scale reflects self-perception and represents the ability to cope with study-related challenges that can lead to stress and anxiety.

Adjustment likely develops over the course of academic studies. The current study examined adjustment at one point in time, and not the developmental aspects of students who continue to study. Future research should explore adjustment as a continuous process in relation to demographic characteristics such as parental support, drug abuse and coping style.

The present study sought to expand the existing literature on students with disabilities in post-secondary education in several ways. First, it examined and compared students with different disabilities to indicate their specific adjustment needs and to see adjustment in relation to visible versus non-visible disabilities. Second, most studies have examined adjustment during the first year of postsecondary education and the current study indicated the persistent adjustment challenges experienced by students with disabilities beyond the first year. Third, the findings indicate that students with mental disabilities and ADHD/LD who all have invisible disabilities, experience significant difficulties in many aspects of post-secondary education. Finally, the results suggest that support services should assess the needs and challenges of students with disabilities and be aware that these may vary among groups of students with different disabilities.

## Data Availability Statement

The raw data supporting the conclusions of this article will be made available by the authors, without undue reservation, to any qualified researcher.

## Ethics Statement

This study was approved by the Research Ethics Committee of the Department of Education at the University of Haifa, Israel. The patients/participants provided their written informed consent to participate in this study.

## Author Contributions

OL and MS conceptualized this study and contributed to the writing and interpretation of the data. IA, AB, AH, and HP contributed to data collection. AB coordinated the data collection of the study. MS contributed to the statistical analysis. All authors agreed to be accountable for the content of the work.

## Conflict of Interest

The authors declare that the research was conducted in the absence of any commercial or financial relationships that could be construed as a potential conflict of interest.
